# Photosynthetic Characteristics of Smaller and Larger Cell Size-Fractioned Phytoplankton Assemblies in the Daya Bay, Northern South China Sea

**DOI:** 10.3390/microorganisms10010016

**Published:** 2021-12-23

**Authors:** Guangming Mai, Xingyu Song, Xiaomin Xia, Zengling Ma, Yehui Tan, Gang Li

**Affiliations:** 1 Key Laboratory of Tropical Marine Bio-Resources and Ecology, South China Sea Institute of Oceanology, Chinese Academy of Sciences, Guangzhou 510530, China; guangingmai@163.com (G.M.); songxy@scsio.ac.cn (X.S.); xiaxiaomin@scsio.ac.cn (X.X.); tanyh@scsio.ac.cn (Y.T.); 2 Southern Marine Science and Engineering Guangdong Laboratory (Guangzhou), Guangzhou 511458, China; 3 University of Chinese Academy of Sciences, Beijing 100049, China; 4 Nansha Marine Ecological and Environmental Research Station, South China Sea Institute of Oceanology, Chinese Academy of Sciences, Guangzhou 510530, China; 5 National and Local Joint Engineering Research Center of Ecological Treatment Technology for Urban Water Pollution, Wenzhou University, Wenzhou 325035, China; mazengling@wzu.edu.cn

**Keywords:** photosynthetic characteristics, cell size, phytoplankton assemblages, Daya Bay

## Abstract

Cell size of phytoplankton is known to influence their physiologies and, consequently, marine primary production. To characterize the cell size-dependent photophysiology of phytoplankton, we comparably explored the photosynthetic characteristics of piconano- (<20 µm) and micro-phytoplankton cell assemblies (>20 µm) in the Daya Bay, northern South China Sea, using a 36-h in situ high-temporal-resolution experiment. During the experimental periods, the phytoplankton biomass (Chl *a*) in the surface water ranged from 0.92 to 5.13 μg L^−1^, which was lower than that in bottom layer (i.e., 1.83–6.84 μg L^−1^). Piconano-Chl *a* accounted for 72% (mean value) of the total Chl *a*, with no significant difference between the surface and bottom layers. The maximum photochemical quantum yield (F_V_/F_M_) of Photosystem II (PS II) and functional absorption cross-section of PS II photochemistry (σ_PS II_) of both piconano- and micro-cells assemblies varied inversely with solar radiation, but this occurred to a lesser extent in the former than in the latter ones. The σ_PS II_ of piconano- and micro-cell assemblies showed a similar change pattern to the F_V_/F_M_ in daytime, but not in nighttime. Moreover, the fluorescence light curve (FLC)-derived light utilization efficiency (α) displayed the same daily change pattern as the F_V_/F_M_, and the saturation irradiance (E_K_) and maximal rETR (rETR_max_) mirrored the change in the solar radiation. The F_V_/F_M_ and σ_PS II_ of the piconano-cells were higher than their micro-counterparts under high solar light; while the E_K_ and rETR_max_ were lower, no matter in what light regimes. In addition, our results indicate that the F_V_/F_M_ of the micro-cell assembly varied quicker in regard to Chl *a* change than that of the piconano-cell assembly, indicating the larger phytoplankton cells are more suitable to grow than the smaller ones in the Daya Bay through timely modulating the PS II activity.

## 1. Introduction

Marine phytoplankton, a polyphyletically diverse group of unicellular primary producers, can produce ~50 Pg C per year by photosynthesis [1]. The cell size of these photosynthetic organisms can span from ~0.6 to >1000 μm in the equivalent spherical diameter from the smallest cyanobacteria to the largest diatoms, with over nine orders of magnitude in biovolume [2,3]. The cell size range of phytoplankton often endows them with advantages in varied marine environments [4]. For instance, small phytoplankton cells that have a high surface-to-volume ratio usually outcompete their large counterparts for growth-limiting resources, e.g., carbon, nitrogen and phosphorus, and thereby dominate in oligotrophic oceans [5,6]. Meanwhile, smaller cells have lower pigment “packaging effect” which enables them to possess a greater light-harvesting ability, and thus grow better under dim-light conditions [7]. On the other hand, these biological traits also make smaller cells more vulnerable to stressful high light or harmful UV radiation if considering the photoinactivation [8] or DNA damage [9]; because, the higher pigment- or volume-specific light absorption usually leads to the higher light-harvesting capacity, as well as the UV exposure per unit pigment or per cell volume [10,11]. The small cell volume also limits the storage of many indispensable resources, e.g., lipids and proteins [12], which may be predicted to count them against surviving in rapidly changing coastal environments.

Light fluctuation usually mediates the photophysiology of phytoplankton. Apart from light intensity or spectrum, the Light:Dark cycle (L:D) can alter the photosynthesis of phytoplankton and, consequently, growth, through varying their light utilization efficiency [13], repair of photoinactivated Photosystem II (PS II) [14], and the amount and activity of photosynthetic enzymes [15,16], as indicated by laboratorial studies. In field conditions, such influences also occur frequently. For example, the photosynthetic performance of phytoplankton assemblages exhibited a clearly light-dependent diel variation in coastal waters of, e.g., the Daya Bay, northern South China Sea [17] and Ariake Bay, Japan [18], as well as in pelagic waters of, e.g., the South China Sea [19], Southern Ocean [20] and Northeast Pacific Ocean [21]. Cell compositions basically mediate such the integrated physiological responses of phytoplankton assemblages to the L:D cycle; while this has been convincingly shown in our laboratorial studies [8,13,14,15,22] and in the work of others [16], there is still a lack of evidence under field conditions.

**Figure 1:**
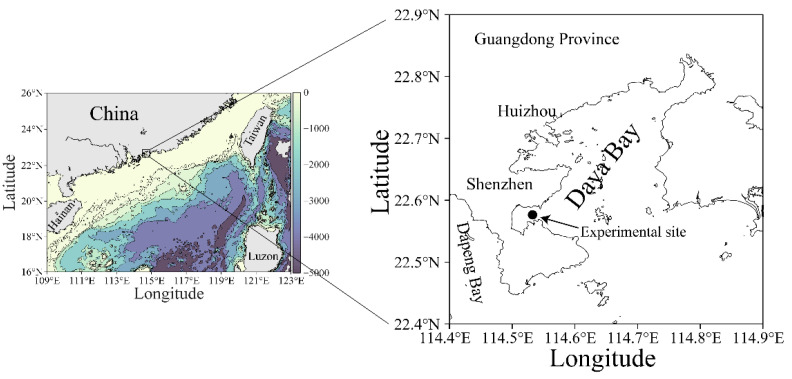
Map showing the experimental site (●) in the Daya Bay, northern South China Sea. The data source of land topography and ocean bathymetry in left map was obtained from NOAA (https://www.ngdc.noaa.gov/, accessed at 20 August 2021).

The Daya Bay is geographically located in a subtropical region of the northern South China Sea (Figure 1). This bay and its adjacent areas have experienced a fast development since the 1980s, making the marine ecosystem therein to be eutrophic [23,24] and resulting in more frequent blooms of harmful algae [25,26]. The underlying physiological clues have been well revealed, too [27,28]. Furthermore, our previous studies showed that phytoplankton assemblages in the Daya Bay exhibited a strong light-dependent diel rhythm in photosynthetic performance [17]. Given the differently biochemical and physiological traits of small and large cells, we hypothesized that the cell-size range of phytoplankton assemblages would alter their photophysiological responses to field environmental changes, and ultimately the diel variation of photosynthetic performance. Therefore, in this study we comparably characterized the photosynthesis of small (<20 µm) and large phytoplankton cell assemblies (>20 µm) with a 36 h in-situ high-temporal-resolution measurement in a coastal water of the Daya Bay. Our studies clarified the differently photophysiological characteristics of small and large natural phytoplankton assemblies, and provided the evidence of cell size-modulated physiological responses to the field light fluctuation.

## 2. Materials and Methods

### 2.1. Study Area

A high temporal resolution experiment was carried out over a period of 36 h with a 2 h interval measurement on 25–26 March 2021 (early spring) in a coastal site (22°35′ N, 114°32′ E) of the Daya Bay, northern South China Sea (Figure 1). This bay is semi-enclosed, with a sprawling coastline of ~52 km, covering an area of ~600 km^2^ with a mean depth of less than 10 m [29]. The tide in this bay is irregularly semidiurnal with surface water-resident time of ~3.2 d [30]. Owing to the ebb and flow of the tide, the depth of the sampling site varied from 3.5 to 4.5 m during the experimental period.

### 2.2. Sampling Protocol

Every 2 h, the natural seawater with phytoplankton assemblages was collected from surface (~0.2 m) and bottom layers (~3.5 m) of the sampling site using a clean 5 L plexiglass water sampler to determine the environmental factors, and biological and physiological parameters described as follows.

#### 2.2.1. Environment Measurements

Every 2 h, the temperature, salinity and pH in surface and bottom layers were measured with a multi-parameter water quality monitor Sonde (YSI 6600, Yellow Springs Instruments, Yellow Springs, USA). Meanwhile, the collected seawater from these two layers were pre-filtered through 0.7 μm pore-sized glass fiber filters (25 mm, Whatman GF/F), and dispensed into 50 mL polycarbonate bottles and immediately stored at −20 °C for later nutrients analysis. After returning to laboratory, the ammonium (NH_4_^+^), nitrate (NO_3_^−^), nitrite (NO_2_^−^), phosphate (PO_4_^3−^) and silicate (SiO_3_^2−^) concentrations were measured using an Automatic Discrete Analyzer (CleverChem Anna, DeChem-Tech. GmbH, Hamburg, Germany), and the N:P ratio was calculated as (NH_4_^+^ + NO_3_^−^ + NO_2_^−^): PO_4_^3−^. The tide height was obtained from the National Marine Information Center of China (http://global-tide.nmdis.org.cn/, accessed at 20 August 2021).

#### 2.2.2. Chlorophyll a (Chl a) Measurement

Every 2 h, 500 mL seawater from the surface and bottom layers were sequentially filtered through 20 μm pore-sized nylon-net filters (25 mm, Merck Millipore Ltd., Carrigtwohill, USA) and 0.7 μm pore-sized GF/F filters. The filters were then wrapped in aluminum foil, immediately frozen and stored at −20 °C for later analysis. After returning to the laboratory, these filters with cell assemblies were extracted overnight in a 5 mL magnesium carbonate saturated 90% acetone (*v*/*v*) at 4 °C in the dark. The extraction was then fluorescently measured with a Turner Designs 10 fluorometer after 10 min of centrifugation at 3500 rpm. Chl *a* concentration was calculated following [31], and the total Chl *a* was calculated by summing these two cell-sized fractions.

#### 2.2.3. Fluorescence Measurement

Every 2 h, 200–300 mL of the collected seawater from surface and bottom layers were filtrated through a 20 μm pore-sized nylon-net filters by gravity. After this, the filtrate was vacuum-filtrated through 0.45 μm pore-sized polycarbonate filters (25 mm, Millipore, Burlington, MA, USA) under the pressure of ~0.2 MPa. Whether this pressure was damaging the cells was not checked under microscopy, although such an effect is limited according to [32]. Phytoplankton cells reposed on the filters were resuspended in 10 mL of 0.45 μm pore-sized filtered seawater to obtain the concentrated large (micro-, >20 µm) and small (piconano-, <20 µm) cell-sized phytoplankton assemblies. Such a separation is operational and used extensively [7,27,28], although the shortcomings are obvious, e.g., some chain-forming species default to micro-cells and some other smaller cells are missing under vacuum. The concentrated samples were then dark-acclimated for 5 min in a 15 mL chamber of fast repetition rate fluorometer (FRRf), coupled to a FastAct base unit (Fast Ocean, Chelsea Technologies Group, Ltd., West Molesey, UK) where temperature was maintained at that of the field conditions, in order to oxidate the electron transport chains and relax non-photochemical quenching (NPQ) [33] before measuring chlorophyll fluorescence. After the dark-acclimation, the FRRf was activated with a single turnover protocol that consisted of 100 saturation flashets of 1 μs duration with 1 μs pitch, followed by 40 relaxation flashets with 60 μs pitch [34]. For each sample, there was a series of 10 actinic light exposures that varied from 0 to 2000 μmol photons m^−2^ s^−1^ and lasted for 60 s for each. The actinic light was delivered from a blue excitation LED (450 nm) in the FastAct instrument. From one to another light exposure step, there was a 20 s dark interval, and the first light step lasted for 120 s as phytoplankton cells need longer time to adapt to the initial transition from a dark to illuminated state [35]. The fluorescence yield from each step was recorded and averaged from 40 consecutive acquisitions. The minimum and maximum fluorescence yields in the dark- (F_O_, F_M_) and light-regulated states (F^’^, F^’^_M_) and the absorption cross-section of PS II photochemistry in the dark (σ_PS II_, nm^2^) and light states (σ^’^_PS II_, nm^2^) were derived from the saturation phase of fluorescence transient with the biophysical model of [34]. The chlorophyll fluorescence was also adjusted by subtracting the fluorescence of GF/F filter-filtrated seawater to eliminate the influence of background fluorescence [36]. Photochemical PS II quantum yields (F_V_/F_M_, F_q_/F^’^_M_) in the dark- and light-state were calculated as [37]:

(1) $$\frac{F_{V}}{F_{M}}=\frac{F_{M}-F_{O}}{F_{M}};\;\frac{F_{q}}{F_{M}^{\prime }}=\frac{F_{M}^{\prime }-\;F^{\prime }}{F_{M}^{\prime }}$$

The fluorescence light curve (FLC)-derived light utilization efficiency (α) and saturation irradiance (E_K_, μmol photons m^−2^ s^−1^) were calculated as [38,39]:

(2) $$\frac{F_{q}}{F_{M}^{\prime }}=\alpha \times E_{K}\times \left(1-e^{-E/E_{K}}\right)\times E^{-1}$$

where E indicates the actinic light.

Then, the maximal relative electron transfer rate (rETR_max_) was calculated as:

(3) $${\mathrm{rETR}}_{\max}=\alpha \times E_{K}$$

### 2.3. Statistical Analysis

All statistical analyses and figures were performed using R software [40]. Nonlinear regression for FLC data were conducted using the “fitWebb” function in package “phytotools” with a Nelder-Mead algorithm [41]. All of the biological and environmental parameters were centered and standardized for redundancy analysis (RDA). The RDA was performed using the “rda” function in package “vegan” to detect the effects of environmental variables on the biological or photophysiological parameters of small and large phytoplankton assemblies [42]. A paired t-test was applied to detect the significant difference, if holding an assumption of normality; otherwise, Wilcoxon signed rank exact test was applied. The significance level was set at 0.05.

In addition, the atmospheric solar photosynthetically active radiation (PAR, μmol photons m^−2^ s^−1^) in the surface (~0.2 m) and bottom layers (~3.5 m) of the experimental site was derived from [43] with an attenuation coefficient of 0.58 m^−1^ in the coastal waters of the South China Sea [44].

## 3. Results

**Figure 2:**
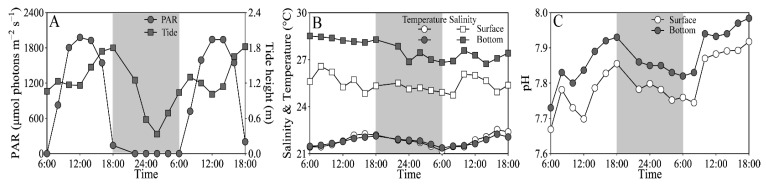
Daily changes in (**A**) photosynthetically active radiation (PAR, μmol photons m^−2^ s^−1^) in the surface (~0.2 m) and bottom layers (~3.5 m) and tide height (m), and (**B**) salinity and temperature (°C) and (**C**) pH in these two layers of the experimental site during 25–26 March 2021. Grey shadows indicate the nighttime. The data of surface PAR, temperature and salinity was derived from our previous publication [43].

On experimental days (25–26 March), the tidal heights of the sampling site ranged from 0.32 to 1.87 m, and the PAR exceeded 1500 and 200 μmol photons m^−2^ s^−1^ at noon-time in the surface and bottom layers, respectively (Figure 2A). The seawater temperature varied from 21.2 to 22.5 °C at the surface, similar to the bottom layer; and the salinity ranged from 24.9 to 26.6 at the surface, being ~10% lower than the bottom layer (Figure 2B). The pH varied from 7.67 to 7.92 at the surface, being significantly lower than the bottom layer (i.e., 7.73–7.98) (t_17_ = 15.64, *p* < 0.01) (Figure 2C). In addition, the dissolved inorganic nitrogen (NH_4_^+^ + NO_3_^−^ + NO_2_^−^), phosphate (PO_4_^3−^) and silicate (SiO_3_^2−^) concentrations that varied respectively from 7.76 to 23.62 μM, from 0.03 to 0.50 μM and from 16.25 to 52.64 μM showed no clear daily change pattern, as well as insignificant difference between the surface and bottom layers (Figure S1).

**Figure 3:**
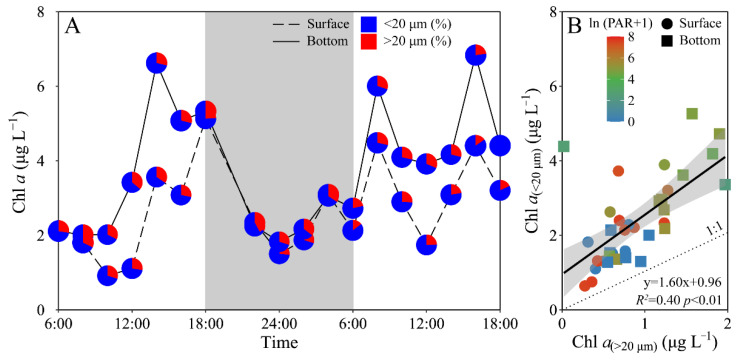
Daily changes in (**A**) total chlorophyll *a* biomass (Chl *a*, μg L^−1^) as well as the allocation (%) of piconano- (<20 μm) and micro-cell-size-fractioned Chl *a* (>20 μm) in surface and bottom layers of experimental site, and (**B**) piconano-cell-fractioned Chl *a* against micro-cell-fractioned Chl *a*. Grey shadow in panel A indicates the nighttime, and the blue and red charts in pies represent the piconano- and micro-Chl *a* allocation. Solid line in panel B shows a linear regression with shadow indicating 95% confidence interval; and the color in symbol indicates the ln(PAR + 1) wherein the PAR is the cell-received irradiance in field condition.

Phytoplankton biomass (Chl *a*) of the experimental site displayed a similar daily variation between the surface and bottom layers, i.e., decreased from morning to midday, and then increased to maximal value at dusk, and again decreased gradually to a minimal value at midnight, followed by an increase to the next morning (Figure 3A). The surface Chl *a* concentration (i.e., 0.92–5.13 μg L^−1^) was significantly lower than that of the bottom layer (i.e., 1.83–6.84 μg L^−1^) (t_17_ = −4.70, *p* < 0.01), especially during daytime. Moreover, piconano-Chl *a* (<20 μm) accounted for 72% (mean value) of the total Chl *a*, with no significant difference between the surface and bottom layers (Figure 3). According to [27], diatoms, e.g., *Chaetoceros socialis*, *Rhizosolenia* sp. and *Nitzschia* sp., and dinoflagellates, e.g., *Scrippsiella trochoidea*, generally dominated the experimental area during the spring and summer periods. 

**Figure 4:**
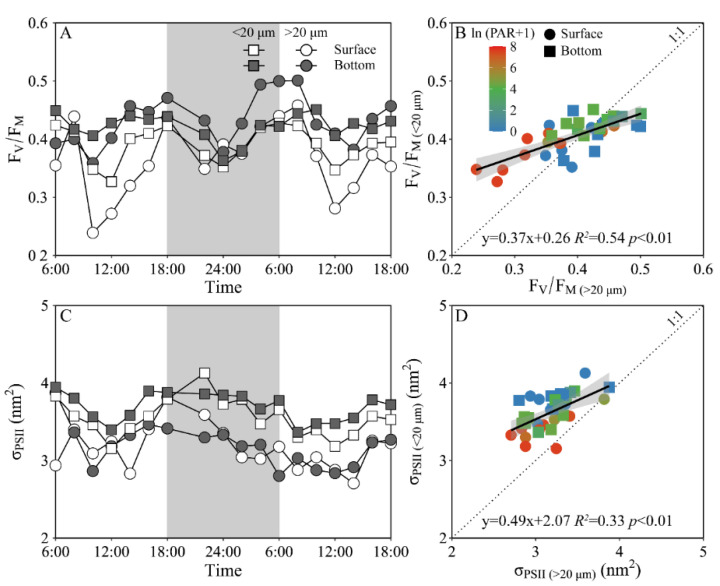
Daily changes in the maximum photochemical quantum yield (**A**, F_V_/F_M_) of Photosystem II (PSII) and absorption cross section for PSII photochemistry (**C**, σ_PSII_, nm^2^) of piconano- (<20 µm) and micro-cell assemblies (>20 µm) in surface and bottom layers of experimental site, as well as the comparisons of (**B**) F_V_/F_M_ or (**D**) σ_PS II_ between piconano- and micro-cell assemblies. Grey shadows in panels A and C indicate the nighttime. Solid lines in panels B and D show the linear regression with shadow indicating 95% confidence interval; and the color in symbol indicates the ln(PAR + 1) wherein the PAR is the cell-received irradiance in field condition.

**Figure 5:**
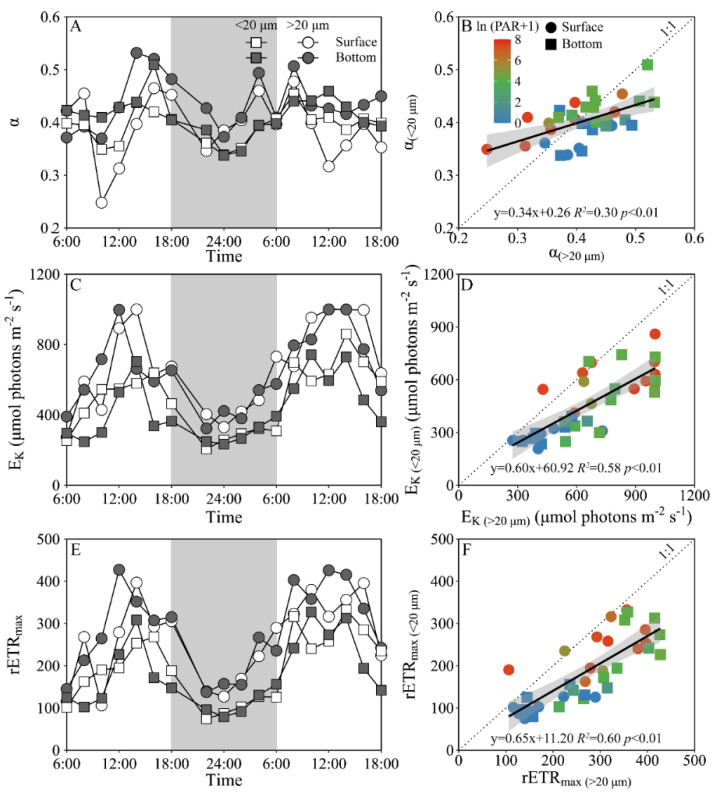
Daily changes in the fluorescence light curve (FLC)-derived light utilization efficiency (**A**, α), saturation irradiance (**C**, E_K_, μmol photons m^−2^ s^−1^) and maximal relative electron transport rate (**E**, rETR_max_) of piconano- (<20 µm) and micro-cell assemblies (>20 µm) in surface and bottom layers of experimental site, as well as the comparisons of (**B**) α, (**D**) E_K_ and (**F**) rETR_max_ between piconano- and micro-cell assemblies. Grey shadows in panels A, C and E indicate the nighttime. Solid lines in panels B, D and F show the linear regression with shadow indicating 95% confidence interval; and the color in symbol indicates the ln(PAR + 1) wherein the PAR is the cell-received irradiance in field condition.

Daily variations of the maximum PS II photochemical quantum yield (F_V_/F_M_) and the functional absorption cross-section of PS II photochemistry (σ_PS II_) of both piconano- and micro-phytoplankton assemblies are shown in Figure 4. The F_V_/F_M_ of the piconano-cell assembly at the surface decreased from 0.43 ± 0.02 to 0.35 ± 0.03 from morning to noontime, then increased to 0.41 ± 0.01 at dusk, and again decreased to 0.37 ± 0.01 at midnight, followed by an increase to next morning (Figure 4A). The F_V_/F_M_ of the micro-cell assembly displayed a greater reduction at noontime than the piconano-ones, indicating the more light-caused photoinhibition. In the bottom layer, the F_V_/F_M_ of both the piconano- and micro-cell assemblies were significantly higher than that of the surface (piconano-, t_17_ = 5.65, *p* < 0.01; micro-, t_17_ = 6.07, *p* < 0.01), in particular at daytime, and showed less daily variation. The σ_PS II_, a measure of quantum yield for PS II photochemistry, showed a similar pattern as the F_V_/F_M_ during daytime; during the nighttime, however, the σ_PS II_ of the micro-cell assemblies from both the surface and bottom layers decreased from dusk until the next morning (surface, *R^2^* = 0.76, *p* < 0.05; bottom, *R^2^* = 0.63, *p* < 0.05), which did not occur in the piconano-cell assemblies (Figure 4C). Moreover, the σ_PS II_ of the piconano-cell assembly that varied from 3.15 to 4.13 nm^2^ was significantly higher than that of the micro-cell assemblies (i.e., 2.71–3.88 nm^2^), no matter in what time and depth (surface, t_17_ = 6.67, *p* < 0.01; bottom, t_17_ = 5.88, *p* < 0.01) (Figure 4D). Consistently, the FLC-derived light utilization efficiency (α) of both types of cell assemblies from both layers showed the same daily change pattern in terms of the F_V_/F_M_ (Figure 5A), while the saturation irradiance (E_K_) and maximal rETR (rETR_max_) mirrored the field light fluctuations (Figure 5C,E). The α of the piconano-cell assembly from both the surface and bottom layers was lower than the micro-cell assemblies under low light or dark conditions (t_25_ = −2.62, *p* < 0.05), but not under high light (Figure 5B); while the E_K_ and rETR_max_ were always lower (t_17_ = 4.37–8.57, *p* < 0.01) (Figure 5D,F).

**Figure 6:**
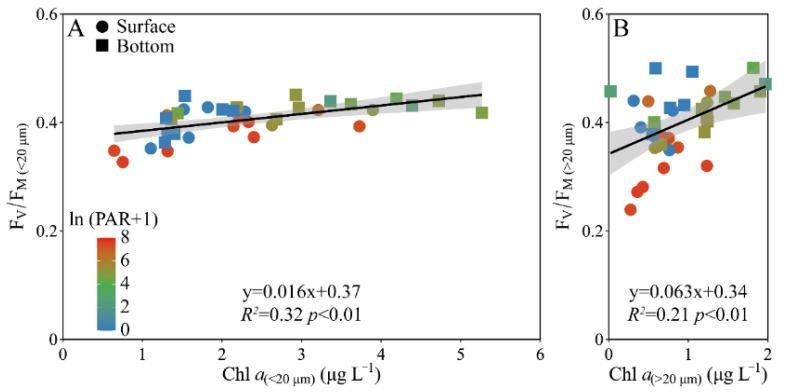
Pairwise relationship between the F_V_/F_M_ and cell-size-fractioned Chl *a* (μg L^−1^) of piconano- (**A**, <20 µm) and micro-cell assemblies (**B**, >20 µm) in surface and bottom layers of experimental site. Solid lines show the linear regression with shadow indicating 95% confidence interval; and the color in symbol indicates the ln(PAR + 1) wherein the PAR is the cell-received irradiance in field condition.

When the pooled F_V_/F_M_ from the surface and bottom layers was plotted against the Chl *a* biomass, a positive correlation occurred in both the piconano- (*R^2^* = 0.32, *p* < 0.01) and micro-cell assemblies (*R^2^* = 0.21, *p* < 0.01) (Figure 6). However, the increased degree, as indicated by the slope, was ~4-fold higher in micro- than piconano-cell assemblies (i.e., 0.063 ± 0.020 vs. 0.016 ± 0.004) (*p* < 0.01), indicating that the large phytoplankton cells are more susceptible to the change in Chl *a* biomass that often mirrors the environmental changes.

**Figure 7:**
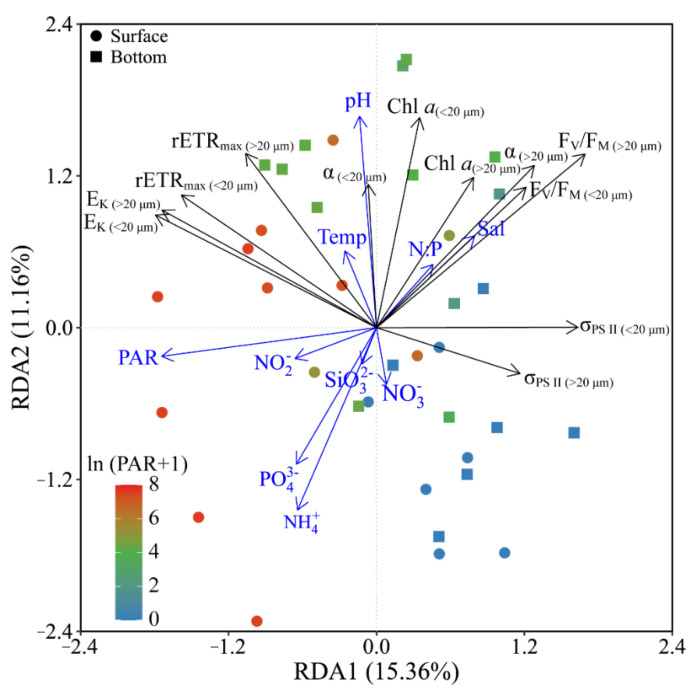
Redundancy analysis (RDA) for correlation of pooled photosynthetic parameters and environmental variables for piconano- (<20 µm) and micro-cell assemblies (>20 µm). Constrained axes RDA1 and RDA2 account for 15.36% and 11.16% of total variance, respectively. The color in the symbol indicates the ln(PAR + 1) wherein the PAR is the cell-received irradiance in field condition. Temp, temperature; Sal, salinity.

Based on the RDA results, phytoplankton biomass (Chl *a*) and photosynthetic parameters (F_V_/F_M_, α and rETR_max_) of both piconano- and micro-cell assemblies were positively correlated to the temperature, salinity and pH, as well as the N:P ratio (Figure 7). Moreover, the F_V_/F_M_, α and σ_PS II_ were negatively correlated to the solar PAR, indicating the photoinactivation of PS II under high light.

## 4. Discussion

Both laboratory and field studies have evidenced that phytoplankton cells exhibit the diel rhythm in photosynthetic behaviors [14,17,19,21]. In this study, we found piconano-phytoplankton cells displayed higher photosynthetic capacity and less photoinhibition under local noontime light, as compared to their micro-cell counterparts. Such a differential degree of the daily variation in photosynthetic parameters between these two differently cell sized phytoplankton assemblies indicated that the cell size range may alter the diel rhythm of their physiological behaviors under field conditions. Moreover, the photosynthetic efficiency of micro-cells varied more quickly to the Chl *a* change than the piconano-cells, suggesting that the larger phytoplankton cells may be more suitable to grow in the variable environments of, e.g., the Daya Bay, through mediating the activity of photosystem in a timely fashion.

Chl *a* biomass in both surface and bottom layers displayed great variation throughout the days (Figure 3), and positively correlated to the tidal heights (surface, *R^2^* = 0.31, *p* < 0.05; bottom, *R^2^* = 0.41, *p* < 0.01). The tidal cycle, i.e., the ebb and flow, is generally believed to alter the movement of the water body, which in turn can change its physio-chemical and biological properties by mixing. Such mixing can also inevitably alter the abundance and species composition of phytoplankton through mixing the water bodies that originally contain different cell biomass and species, and/or through altering the physio-chemical properties and thus the cell growth [45,46]. The tide rise-induced seawater intrusion often brings more clear water into the sampling site, and as a result the phytoplankton within this water would be higher if considering the presence of more underwater solar energy and plentiful nutrients (Figure S1) [23] that would endow them to grow faster. Therefore, the seawater intrusion may have contributed to the higher Chl *a* biomass (Figure 3A). What is more, the clearer water body in the sampling site during high tide may also benefit the growth of phytoplankton cells, but this effect on the Chl *a* biomass would be eliminated in such an hourly scale. The nutrients concentration also plays a pivotal role in modulating phytoplankton growth and even blooms [47], but such a nutrient-induced activation for cell growth could not explain the Chl *a* being so altered in an hourly scale either [48]. Finally, the small cell-sized phytoplankton prevailed in the experimental area, consistent with previous studies [17,27,28]. It seemed to be contradicted that larger cells can regulate the PS II activity faster than smaller ones (Figure 6) and are thus more suitable to grow in the varied environments of coastal regions. This may be explained that the sampling site is located in shellfish culture areas [49], and the selective feedings of shellfish may have altered the community structure of phytoplankton assemblages [49,50].

The photosynthetic parameter of F_V_/F_M_ usually decreases under stressful high light, caused by the photoinactivation of PS II [13,51,52]; therefore, it is often used as an indicator of photosynthetic capacity of phytoplankton cells [8,17]. Consistently, the F_V_/F_M_ was lower at noontime than the other times (Figure 4A); while this decline was less in piconano- than in micro-cell assemblies, which, together with a similar decline of the FLC-derived light utilization efficiency (α, Figure 5A), implies that smaller cells are more resistant to high light. However, this finding is contrary to the previously-established concept that the lower package effect of smaller cells can render them more susceptible to high light [8,53]. Such a contradiction may be explained by the fact that the smaller cells have higher damage repair capacity, and, considering their higher metabolic rate, they may have actively energized the repair process under stressful high light [52], thus maintaining the higher photosynthetic efficiency than their larger counterparts (Figure 4A and Figure 5A). Moreover, smaller cells often have higher antioxidant activity that may help them to relieve the oxidative damage triggered by reactive oxygen species (ROS) [16], thus enhancing their high light resistance. More interestingly, our results also showed that the F_V_/F_M_ of the piconano-cell assembly varied more slowly to the Chl *a* biomass change than the micro-cell assembly (Figure 6), which indicates the slower reactiveness of smaller cells to environmental changes, and by contrast, the larger cells can adjust to the activity of PS II in a timely fashion and may be thus more suitable to grow in the variable environments of the Daya Bay. This was also supported by the higher changing amplitude of saturation irradiance (E_K_) and electron transport rate (rETR_max_) in micro-cell assembly than in piconano-cell assemblies (Figure 5C,E). Furthermore, the higher σ_PS II_ of piconano-cell assembly than micro-cell assemblies (Figure 4C,D) indicates the higher light-harvesting ability of smaller cells, also supporting their higher light utilization efficiency (Figure 5A) and photosynthetic rate (Figure 5E). It may benefit them in turbid water too, thus maintaining their high abundance in the Daya Bay, as found in this study (Figure 2) or in others [17,27,28].

## 5. Conclusions

In this study, we found that the tide’s coming in and going out co-varied with the biomass and community structure of phytoplankton assemblages from the experimental site through moving the water bodies. We also found the piconano-phytoplankton assembly exhibited higher photosynthetic efficiency, as well as more tolerance to high light compared to their micro-cell counterparts. Moreover, we found the piconano-cell assembly exhibited less daily variation in the photosynthetic parameters and slower physiological reactiveness to environmental changes, as compared to micro-cell assemblies. In particular, our results from in-situ conditions, together with those from laboratory conditions [13,14,22], indicated that the varied community structure modulates the diel rhythm of the photophysiological behaviors of phytoplankton assemblages in the Daya Bay. In addition, our findings in the eutrophic regions may have been tempered by considering that the concentration of the 0.45 µm pore-size filters under vacuum for piconano-cell assembly may not cover all smaller cells of, e.g., *Synechoccocus*, and such a tempered effect would be even more evident in the piconano-cell dominated oligotrophic oceans [7] or summer season of the Daya Bay [27]. Therefore, further studies should be performed in other nutrient-status regions or other seasons to generalize an implication being suitable for broader frames.

## Data Availability

The data presented in this study are available within the article.

## Supplementary Materials

The following are available online at https://www.mdpi.com/article/10.3390/microorganisms10010016/s1, Figure S1: Daily changes in concentrations (μM) of ammonium (A, NH_4_^+^), nitrite (B, NO_2_^−^), nitrate (C, NO_3_^−^), silicate (D, SiO_3_^2−^), phosphate (E, PO_4_^3−^) and N:P ratio (F) in surface (~0.2 m) and bottom (~3.5 m) layers of experimental site during 25–26 March 2021. Grey shadows indicate the nighttime.
