# Supplementary material for: Photosynthetic Characteristics of Smaller and Larger Cell Size-Fractioned Phytoplankton Assemblies in the Daya Bay, Northern South China Sea
Source: Microorganisms. 2021 Dec 23;10(1):16. doi: 10.3390/microorganisms10010016 (PMC8846320; doi:10.3390/microorganisms10010016)

Supplemental Figure

Figure S1: Daily changes in concentrations (μM) of ammonium (A, NH_4^+), nitrite (B, NO_2^-), nitrate (C, NO_3^-), silicate (D, SiO_3^{2-}), phosphate (E, PO_4^{3-}) and N:P ratio (F) in surface (~0.2 m) and bottom (~3.5 m) layers of experimental site during March 25-26 of 2021. Grey shadows indicate the nighttime.

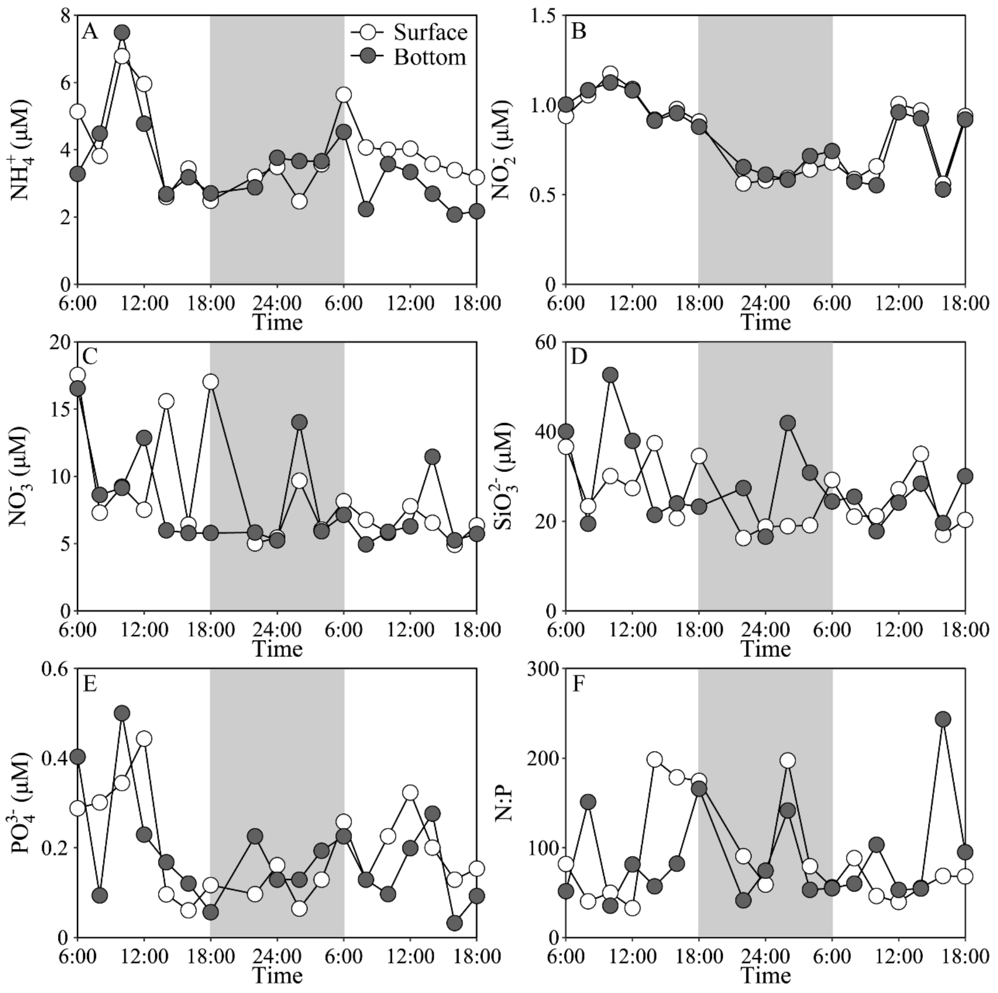

Supplement: Supplementary file 1 [file microorganisms-10-00016-s001.zip › microorganisms-1529040-SI.pdf]
